# Optical Interconnects Finally Seeing the Light in Silicon Photonics: Past the Hype

**DOI:** 10.3390/nano12030485

**Published:** 2022-01-29

**Authors:** Hosam Mekawey, Mohamed Elsayed, Yehea Ismail, Mohamed A. Swillam

**Affiliations:** 1 Center for Nanoelectronics and Devices (CND), The American University in Cairo, Cairo 11835, Egypt; mohamed_elsayed@aucegypt.edu (M.E.); y.ismail@aucegypt.edu (Y.I.); 2 Faculty of Mathematics and Computational Sciences, University of Prince Edward Island (UPEI)-Cairo Campus, Universities of Canada in Egypt, Cairo, Egypt; 3 Physics Department, American University in Cairo, Cairo 11835, Egypt; m.swillam@aucegypt.edu

**Keywords:** optical interconnects, silicon photonics, electrooptic modulators, slot waveguide, grating coupler, semiconductor laser, hybrid integrated circuits, Complementary Metal Oxide Semiconductor (CMOS) compatible, pin photodetector, avalanche photodetector, plasmonic, nanophotonics, photonic integrated circuits

## Abstract

Electrical interconnects are becoming a bottleneck in the way towards meeting future performance requirements of integrated circuits. Moore’s law, which observes the doubling of the number of transistors in integrated circuits every couple of years, can no longer be maintained due to reaching a physical barrier for scaling down the transistor’s size lower than 5 nm. Heading towards multi-core and many-core chips, to mitigate such a barrier and maintain Moore’s law in the future, is the solution being pursued today. However, such distributed nature requires a large interconnect network that is found to consume more than 80% of the microprocessor power. Optical interconnects represent one of the viable future alternatives that can resolve many of the challenges faced by electrical interconnects. However, reaching a maturity level in optical interconnects that would allow for the transition from electrical to optical interconnects for intra-chip and inter-chip communication is still facing several challenges. A review study is required to compare the recent developments in the optical interconnects with the performance requirements needed to reach the required maturity level for the transition to happen. This review paper dissects the optical interconnect system into its components and explains the foundational concepts behind the various passive and active components along with the performance metrics. The performance of different types of on-chip lasers, grating and edge couplers, modulators, and photodetectors are compared. The potential of a slot waveguide is investigated as a new foundation since it allows for guiding and confining light into low index regions of a few tens of nanometers in cross-section. Additionally, it can be tuned to optimize transmissions over 90° bends. Hence, high-density opto-electronic integrated circuits with optical interconnects reaching the dimensions of their electrical counterparts are becoming a possibility. The latest complete optical interconnect systems realized so far are reviewed as well.

## 1. Introduction

**Figure 1:**
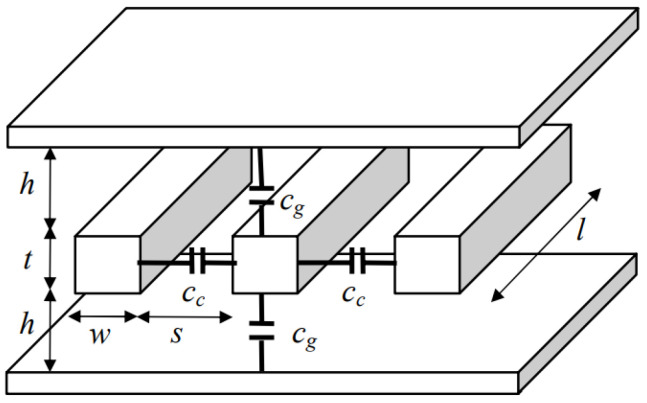
Capacitance between neighboring wires (*_Cc_*) and adjacent metal layers (*C_g_*) [32], © 2022 IEEE. Reprinted with permission, from M. Ghoneima, Y. Ismail, M. M. Khellah, J. Tschanz, and V. De, “Serial-Link Bus: A Low-Power On-Chip Bus Architecture,” IEEE Transactions on Circuits and Systems I: Regular Papers, vol. 56, pp. 2020–2032, 2009.

The interconnect problem in the integrated circuits industry was brought up a couple of decades ago [1] and the search continues for a solution [2,3,4,5,6,7,8,9,10,11]. Global interconnects that generally span the chip size do not benefit from scaling as chip sizes remain approximately constant or even increase [12]. To be able to keep up with increasing bandwidths and more stringent power requirements, different materials have been adopted, but further advancement has been hampered by integration challenges [13]. Local interconnects, on the other hand, have traditionally benefited from scaling as wires became shorter. However, with decreasing device geometries and increasing circuit functionality and complexity, wire density presents a serious bottleneck. To be able to pack more wires into less space, wire width decreased, which increased their resistance. To mitigate this, the height of the wires has been increased to reduce the resistance, but this comes at a tradeoff with the increased capacitance between the wires as they can become closer to each other [6], as shown in Figure 1. With nanoscale wires, large fabrication variability has drastically increased [13,14].

The signal coupling has also surfaced as an important issue, where a signal in one wire may undesirably couple to a neighboring wire, adding noise [5,6,7,8,9,10,11,12,13,14,15,16,17,18]. In the meantime, these problems have been mitigated by the judicious use of repeaters [19,20,21] and wire-aware designs [20,21,22], such as making sure that components that communicate with each other frequently are close together to reduce delays and keeping wires with frequent activity far away from each other to reduce coupling noise [2,4,23]. Passive and active shielding have been employed to solve the signal coupling issue [2,4,23]. Some promising solutions are actively being researched to keep metal wires in chips for longer. Balasubramonian et al. proposed to use different wires for different purposes, i.e., optimizing for latency, bandwidth, or power [24].

Signaling using a low voltage swing was proposed [25,26] as a possible solution in metal interconnects, but it has many problems in practice because of noise sensitivity and associated sensitive analog circuitry. Ghoneima et al. and others have been working on serialization–deserialization protocols, which have breathed life into metal interconnects by enabling the long wires to work as transmission lines and benefit from the speed of light transmission speeds at low power levels [27,28,29,30,31,32], but we are already very near the theoretical limits, whether this be for wire density or power consumption [33]. The search for a complementary technology of choice for interconnects remains of paramount importance.

### 1.1. Interconnect Bottleneck in Chip Multiprocessors (CMPs)

Moore’s law will continue to use many-core chips. Future processors will contain hundreds or even thousands of cores communicating with each other through a network on chip (NoC). The NoC used to communicate between the different cores is a hot topic [34,35,36]. Many-core chips using state-of-the-art metal wire technology, including repeaters and low voltage swings, will still find their whole power budget (150 W to 200 W) taken up by interconnects [5]. Another power management technique is to turn off parts of the chip to avoid overheating. The parts of the chip that are turned off to reduce power consumption, so-called “dark silicon”, are growing at an alarming rate that limits multicore scaling [37]. Interconnects currently consume half a microprocessor’s power and it is only expected to increase, reaching 80% of the total microprocessor power [22].

The interconnect problems highlighted earlier are exacerbated even more in supercomputers. Moving towards exascale supercomputers capable of performing 10^18^ operations per second, reliability is a great issue with no real solution in sight [38]. There is an immense need to improve efficiency as exascale supercomputers are predicted to have electricity bills in the hundreds of millions of dollars per year, and the traditional voltage scaling will not work because it will add to the reliability problem.

Neither the metal wires nor the transmission lines can keep up with the ever-increasing bandwidth requirements that will exceed 100 Gbps for manycore processors [39,40,41]. The optical interconnects, on the other hand, have already surpassed 100 Gbps and are theoretically able to go beyond 1 Tbps using dense wavelength division multiplexing methods [39]. As we shall see, there is no free lunch. While optical interconnects bring about an exciting future, many challenges need to be overcome, mostly with respect to power consumption and integration. Progress has been very rapid to overcome these challenges over the past few years, so we can be optimistic.

### 1.2. Optical Interconnects

Optical interconnects are not the only proposed solution to the interconnect bottleneck [39]. Wireless network-on-chip suffers from a decaying signal with long distances and a limited bandwidth [42,43]. Therefore, there are concerns about its long-term viability as a technology of choice. We also briefly mentioned transmission lines; they will not be able to solve the problem for too long as there are issues with the scalability [39,40,41,44].

Many researchers have highlighted the strong need for optics in supercomputing [45,46]. In addition to solving the interconnect problems, there are approaches for ‘zero-energy’ logic and calculations using passive optical components that could be game-changers in supercomputing due to the heating problems [46]. Researchers at IBM have highlighted the importance of optical interconnects in supercomputing [45]: “Interconnects of the future will be dominated by optics, as this offers the potential for a far better cost solution for all distances. As we look to exascale, even the connections between processors on a common circuit card need to be optical because of the amount of bandwidth needed. Silicon photonics represents a nearly ideal solution to the interconnect problem.”

By now, it is clear that electrical interconnects are posing the bottleneck for further advancements in many-core systems and exascale supercomputers. The theoretical limits associated with optical interconnects mean that it will eventually bring in endless possibilities and will continue to advance with technology scaling. Questions, such as “Why do we need optical interconnects?” or “Are optical interconnects the solution to the interconnect bottleneck?”, are no longer important because a strong case has been made [33,46]. To date, metal interconnects are still the technology of choice because they are the cheapest and simplest way to route signals. However, with the first one-thousand-core processor unveiled [47], it is becoming imperative to make the switch. The most relevant question now is: what needs to be done for optical interconnects to be ready when the decision to switch is made?

### 1.3. Performance Requirements for Optical Interconnects

**Figure 2:**
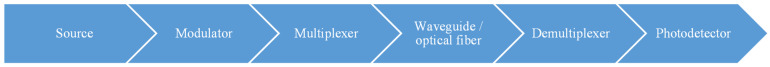
Data propagation through an optical interconnect system. Continuous-wave light is generated from the source. Data is transferred from the electrical domain to the optical domain using the modulator. The data-carrying capacity of an optical fiber usually far exceeds that of copper, thus it is possible to combine data onto one optical fiber using multiplexing methods. On the receiving end, the light passes through a demultiplexer, then a photodetector is used where the data is recovered to the electrical domain.

Figure 2 shows a schematic of the components that comprise an optical interconnect system.

This review is concerned with optical interconnects for multicore and many-core systems. Therefore, silicon photonics plays a very important role. There is generally a schism within the circle of researchers working with silicon photonics: one group considers integrating non-silicon components with silicon as silicon photonics (albeit “hybrid” or “heterogeneous integration”), even if this consists of changes in standard wafer processing. The other group is adamant on “zero-change”, i.e., using the same fabrication flow that is currently used with electronics to build photonics circuits. Each approach has its advantages and disadvantages.

The requirements laid out about a decade ago, largely remain accurate [33] and are summarized as follows: off-chip optical interconnects should consume 100 fJ/bit or lower, this number will be reduced to 50 fJ/bit by 2022. On-chip optical interconnects should be 50–200 fJ/bit, and by 2022 that number should be 10–30 fJ/bit. The total bandwidth of all interconnects on a chip is expected to reach 780 Tbps by 2022.

Heterogeneous integration has been able to demonstrate components that meet the device requirements for optical interconnects, but we have yet to see a complete optical interconnect system that meets these requirements. Zero-change silicon photonics, on the other hand, are still far behind; complete systems consume around several pJ/bit and the bandwidth has not reached 10 Gbps [48,49]. This is because the rigid rules imposed by the zero-change process leave little flexibility for designers. In both cases, the advancements in the fabrication will definitely translate to performance improvements. As discussed in the heterogeneous integration road map for 2021, there are different integration platforms for photonics: chip-level integration that is slow and expensive, wafer-level integration with its cost expected to decrease with maturity, and system-in-package (SiP) integration, which offers the highest functional density and performance at lower latency, cost, and power. The challenges facing heterogeneous integration include, thermal management and test access. The possible potential solutions are discussed in [50].

The remainder of the paper is divided as follows. Section 2 discusses the individual components of an optical interconnect system at length, starting with (A) optical sources and (B) different waveguide platforms, then (C) modulators and ending with (D) photodetectors. Recent advances in multiplexing/de-multiplexing optical devices are reviewed in detail in [51,52,53]. In Section 3, the complete interconnect systems recently demonstrated will be highlighted. Section 4 discusses the future outlook of optical interconnects and concludes the paper.

## 2. Components of an Optical Interconnect

**Figure 3:**
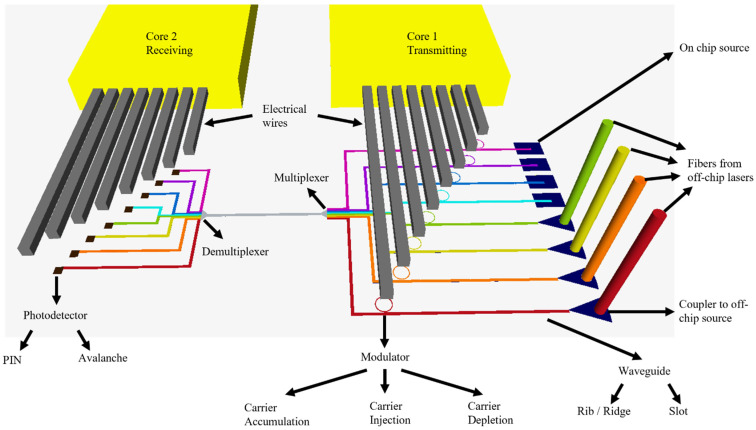
3D schematic of the optical interconnect between core 1 and core 2. Light is introduced to the system by either coupling off-chip lasers using grating couplers or by using an on-chip source. The different colors represent the different wavelengths, where each wavelength is considered as a data channel. The electrical data from core 1 is transmitted through metal layers to the electro-optical modulator, which converts the data from the electrical domain to the optical domain. Wavelength division multiplexing is used to increase the capacity of the system. A multiplexer combines the data from different wavelengths into one waveguide. For core-to-core communication on the same chip, simply an optical waveguide will suffice. For long-distance communication, the data can be transferred to an optical fiber using coupling methods, such as surface grating couplers or edge couplers. A demultiplexer splits the data back into their channels, and a photodetector converts the data back into the electrical domain. The schematic shows the various alternatives for the source (off-chip/on-chip), waveguide (strip/slot [63]), modulator (carrier accumulation/carrier injection/carrier depletion), and photodetector (PIN/avalanche).

Figure 3 summarizes the components for an optical interconnect system that will be discussed in detail in this paper. There are three major components in addition to waveguide routing and multiplexing techniques.

### 2.1. Source

There are two methods to introduce light into the system. The first one is to couple light from an off-chip source to the chip. The second approach is to have an on-chip source.

#### 2.1.1. Off-Chip Sources

There are a variety of advantages to having off-chip sources. The stability of off-chip sources is much easier accomplished than on-chip sources. First, the heat generated by lasers can be 3 to 10 times the optical power of the laser [54]. Thus, the thermal management of this heat can complicate the on-chip interconnect system in multiple ways. On the other hand, the buried oxide used as cladding is a thermal insulator; thus, off-chip heat sinking is more effective. In addition, stable laser operation requires a constant temperature and the whole chip would have to be cooled. Second, as will be explained in-depth shortly, III–V lasers are more mature than silicon lasers due to their higher efficiency, resulting in high yields and commercial availability.

The historical developments leading to choosing silica as the standard material for optical fibers are reviewed in [55]. In any case, this material choice leads to the mode field diameter for a single-mode fiber being between 6 and 10.4 µm [56]. On the other hand, for a height of 220 nm, the typical single-mode silicon waveguides are 150 nm to 400 nm in width [54] leading to losses above 30 dB, which is obviously unacceptable [57]. This mode mismatch needs to be solved. Table 1 compares the different types of packaging solutions for coupling light from the optical fiber to the silicon photonic waveguide.

In Table 1, the operation wavelength is 1550 nm, while the 1 dB bandwidth refers to the range of wavelengths around the operation wavelength for which the drop in transmission is less than 1 dB.

The larger this bandwidth, the better, as the coupler can be used in wavelength division multiplexed systems for large transmission capacity, in which each wavelength is a data channel carrying a different set of data. A narrow bandwidth is suitable in case only the 1550 nm wavelength is used. In case a narrow bandwidth is used and a high data-carrying capacity is required, stringent requirements for the lasers will be needed as the linewidth of the laser has to be as narrow as possible.

The remainder of this section will discuss the methods for efficiently coupling light from off-chip sources to the chip. This is accomplished either through optical fibers or co-packaging the laser on the chip. In both cases, this coupling can be performed by using grating couplers or by edge-coupling.

##### Edge-Coupling

Edge-coupling has not been used in complete interconnect systems due to the non-standard fabrication involved, but deserves attention because of its advantage in broadband systems. Edge-coupling between a fiber and silicon photonic waveguide is typically performed by using a spot-size converter, as shown in a. The advantage in comparison to grating methods is that it allows broadband transmission.

The fabrication requires depositing materials with a refractive index between that of silicon dioxide (*n* = 1.44) and silicon (*n* = 3.47), and can either be typically silicon nitride [56] or a polymer [57,64,65], and in all cases losses can be below 1 dB in perfect alignment conditions. While CMOS compatible, the thickness of the spot size converter is generally not required for any other part of the chip and is thus an added expense in the fabrication process. Another important limitation is that the placing of the fiber has to be at the edges of the chip only, severely limiting the number of IOs on a chip. Additionally, edge coupling requires a post-fabrication processing: the accurate dicing and polishing of the edge of the chip is needed to reduce the scattering/reflections. This is not realized with lithography precision and large fabrication variability will exist between the different chips. The use of a lensed fiber, as shown in a, better matches the modes for higher efficiency and has been applicable for high optical power (around 1 W) [58]. However, fabrication tolerance is very tight, as shown in Table 1, and the permanent welding of the fiber to the chip is justified using materials that are resistant to thermal expansion.

##### Vertical Grating Couplers

**Figure 4:**
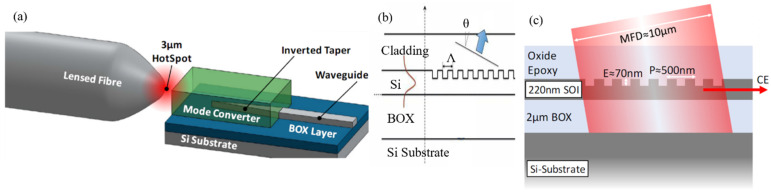
(**a**) Edge coupler utilizing a spot size converter [58]. Reproduced under CC BY. (**b**) Cross-section schematic of the grating coupler [54], © Cambridge University Press 2015. Adapted by permission from Cambridge University Press: L. Chrostowski and M. Hochberg, silicon photonics design: from devices to systems. (**c**) Cross-section schematic of a grating coupler [58]. Reproduced under CC BY.

Figure 4b is used to explain the operation principle. It is based on Bragg diffraction and is explained in detail in [54]. Briefly, light with a wavelength λ from a waveguide diffracts when it reaches the diffraction grating. A fiber placed on top of the gratings will collect this light if the waveguide propagation constant β in (1)

(1) $$\beta =\frac{2\pi n_{eff}}{\lambda }$$

is matched with the horizontal component of the diffracted light $k_{x}$ described by the Bragg condition in (2)

(2) $$\beta -k_{x}=m\cdot \frac{2\pi }{\Lambda }$$

where $n_{eff}$ is the effective refractive index of the waveguide, m is the diffraction order, and Λ is the period. The diffracted light is shown by the blue arrow in Figure 4b. An important design parameter is the angle θ at which the light diffracts, as is described by (3):

(3) $$n_{eff}-\sin\theta =\frac{\lambda }{\Lambda }$$

while the theory of operation is described for light traveling from the waveguide to the fiber, it is a reciprocal system so that the same design will focus light from the fiber to the waveguide.

Figure 4c shows a cross-section schematic of a vertical grating coupler. Vertical coupling gratings have the advantage that no post-fabrication steps are needed and only a 3 dB to 0.9 dB insertion loss is achievable [66,67]. Another great advantage is that coupling can be performed anywhere on the chip. An interesting approach is to co-package the laser with the chip without using a fiber [68]. The main disadvantage of grating couplers, however, is that it is optimized for a specific central wavelength so it is generally a short free spectral range of only 35 to 80 nm in wavelength. Therefore, wideband systems cannot use a vertical coupling grating. However, for interconnect applications, such a wideband operation is usually not required even with dense wavelength multiplexing. This is why vertical grating couplers have been the method of choice for complete interconnect systems [69,70]. Luxtera employed a holographic lens system to couple light to its on-chip optical systems [71]. It is also feasible to align many (hundreds) fibers to many couplers simultaneously using an “optical shunt” [58]. Additionally, larger gratings allow more relaxed alignment tolerance (±10 um for >1 dB loss) at the expense of an even narrower bandwidth [58].

For permanent fixtures, a problem with vertical grating couplers is the mechanical robustness of the fiber packaged on top of the grating coupler. Teraxion pioneered a new approach—surface grating coupler—complementing both vertical grating couplers and edge couplers. By clever engineering, the grating period can be designed so that the diffraction angle is almost 0. Thus, the fiber is placed parallel to the chip with a very small insertion angle [72].

An interesting innovation dubbed meta-material gratings involves the sub-wavelength patterning of silicon gratings (with dimensions often below 100 nm), enabling the engineering of the effective refractive index of the material used in the grating coupler [73]. This additional degree of freedom can offer lower insertion loss and a higher bandwidth [58].

#### 2.1.2. On-Chip Sources

Photonic packaging is far more expensive than electronics packaging and is described as “perhaps the most significant bottleneck” in integrated photonic device development [58]. By using on-chip interconnects, all light generation and detection will be performed on-chip, and photonic packaging will no longer even be necessary. The yield when using on-chip sources is predicted to exceed off-chip sources, as the coupling method is very sensitive to inaccurate post-fabrication modifications.

Efficient lasing in silicon is very challenging because it is an indirect band gap semiconductor. This section first starts with a brief review of the attempts at overcoming these challenges in silicon. Another arguably more promising approach is the heterogeneous integration with III–V on-chip sources. Table 2 summarizes this section.

##### Lasing in Silicon

Quantum confinement effects can also cause light emission in silicon. Different structures exhibiting quantum confinement have been demonstrated to emit and/or amplify light and can be categorized as 1D structures (nanowires, nanopillars) and 0D structures (porous silicon, nanocrystals). Due to the obvious interaction with phonons when emitting light in silicon, heat has a very critical effect. Early reports of lasing in silicon demonstrated emitting different wavelengths of light at cryogenic temperatures [74]. Lasing using nanocrystals, on the other hand, was demonstrated at room temperature by amplified spontaneous emission; however, this required an external source of light [75]. In addition, to avoid the critical heat issues, only very short pulses of light were possible.

Raman scattering has been used to generate light in silicon-based on an external optical pump. Two-photon absorption (TPA), due to the long recombination time of photogenerated carriers, has been a barrier to high transmission [76,77]. A reverse-biased PN junction was used in a silicon gain cavity to sweep away the carriers and overcome free carrier absorption in all-silicon Raman lasers reaching an output power of 50 mW [76,77]. Efficiency however is limited at 28%. Due to such a low efficiency, Raman lasers have not been demonstrated in complete optical interconnect systems. Another problem was the prohibitively large size of the gain cavity, requiring a length that could reach several centimeters even when a ring laser cavity was used [77].

Another option for lasing in silicon relies on blackbody radiation. When heated, silicon emits infrared radiation and it may be possible to use this as a source of light in mid-infrared applications [78,79].

##### Heterogeneous Integration

Despite many reports at the beginning of the century predicting the end of silicon [80,81], the IC industry was reluctant to incorporate different materials over the past few decades. However, heterogeneous integration is imminent in recent ITRS reports (2014), especially with III–V semiconductors. Hence, large-scale hybrid devices, such as InP/Si lasers and Ge/Si photodetectors, can become a reality in the not-too-distant future.

As expected, the non-silicon lasers easily outperform silicon lasers because they can have a direct bandgap and they do not suffer from two-photon absorption. InAs quantum dots grown on silicon have been shown to operate up to 110 °C temperatures, thus they are compatible with the harsh environment often found in electronic ICs [82]. Furthermore, they exhibit an efficiency of 37% and output power above 176 mW, numbers unheard of with silicon [82]. The same lab in UCSB more recently added a GaAs buffer between the silicon and InAs quantum dots and achieved a higher rate of power and efficiency. More importantly, reliability was key in this work with extrapolated mean-time-to-failure of millions of hours, ensuring the commercial viability of this technology [83]. Chen et al. also demonstrated reliable lasers based on InAs quantum dots on silicon with extrapolated mean-time-to-failure of over 100,000 h and operation at temperatures of up to 120 °C [84].

Heterogeneously integrating III–V gain media on the silicon offered advantages that were not possible by only using III–V media. A limiting factor when using III–V material is that it is used to generate the photons; spontaneously generated photons can cause free carrier generation that can attenuate the gain. By directly placing the gain material on top of the silicon, the photons were quickly swept to the silicon and thus did not cause any problems in the gain section [85].

Wavelength division multiplexing (WDM) is the main driver behind the high bandwidth possible in optical interconnects. Each wavelength is a data channel, and the waveguide is able to carry light at all the different wavelengths while maintaining the integrity of the data contained in each channel. This enables a single waveguide to carry the data that would normally be carried using dozens of copper wires. To enable dense wavelength division multiplexing, the linewidth of the laser is a very important parameter; narrower linewidth allows denser multiplexing [85].

**Figure 5:**
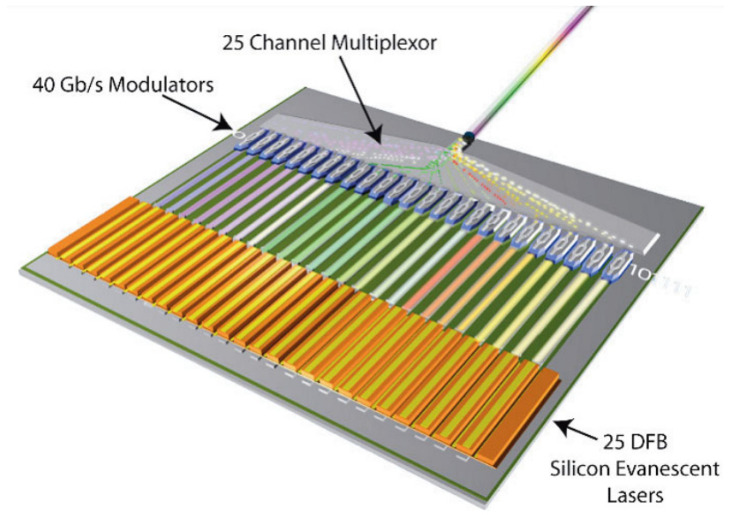
Schematic showing the concept of an integrated 1 Tbps hybrid silicon photonic transmitter [93]. The 25 lasers should have a very narrow linewidth to allow using a narrowband photodetector, which can be simpler and more efficient than a wideband photodetector. Copyright © 2022 WILEY-VCH Verlag GmbH & Co. KGaA, Weinheim. Reprinted by permission from John Wiley and Sons: B. R. Koch, A. W. Fang, E. Lively, R. Jones, O. Cohen, D. J. Blumenthal, and J. E. Bowers, “Mode locked and distributed feedback silicon evanescent lasers,” Laser and Photonics Reviews, vol. 3, pp. 355–369, 2009.

Figure 5 shows how the output of 25 lasers, each operating at a different wavelength, can be modulated and wavelength-multiplexed to create a 1 Tbps silicon-based transmitter [93]. Liu et al. demonstrated how the narrow linewidth of 1.8 kHz can be used to transmit 4.1 Tbps using 64 channels [94].

##### External Cavity Lasers

An external cavity laser is composed of two chips: an active chip, typically formed by III–V reflective semiconductor optical amplifiers butt-coupled to a silicon waveguide, and a silicon photonics circuit designed to be wavelength-selective, which may involve bragg reflectors [95], ring resonators [96], or photonic crystals, for example [95]. The space in between these two chips is the external cavity. Over the past decade, the linewidth achieved by external cavity lasers decreased from around 100 kHz to ~1 kHz [97] and even sub-kHz by choosing Si_3_N_4_ as the waveguide material [98]. The narrowest laser linewidth of 40 Hz was achieved using an external cavity laser using InP active material and an Si_3_N_4_ photonic circuit involving a spiral waveguide, 3 micro ring resonators, and a Sagnac mirror [90].

### 2.2. Waveguide

The basic waveguide is considered as the platform upon which the optical interconnect system is built. A high index core allows for smaller waveguide pitch and tighter bends, while a low index core offers lower propagation delay [99]. Optical waveguides offer a better propagation delay than any electronic interconnect. The current trends show that optics will migrate from board levels to the chip level, within the next few years [100]. However, the size mismatch between the optical waveguides and the existing electrical interconnects poses a challenge for on-chip optical interconnects integration [100,101,102]. Additionally, due to the size of the guided mode footprint, usually a 0.5 to 3 µm separation distance is required between the adjacent waveguides to avoid crosstalk. This, in turn, impacts the bandwidth density that can be realized. Wavelength division multiplexers (WDMs) can be utilized to enhance the I/O bandwidth [99]. The most common silicon waveguide is a strip waveguide with a height of 220 nm, built in the top device layer in typical SOI wafers. For single-mode propagation, widths of 400 nm to 500 nm are used, with losses of around 0.1 to 0.3 dB/mm [71,103]. As soon as such low losses in silicon waveguides were demonstrated, silicon photonics as an industry boomed. Silicon nitride waveguides are also considered as a very attractive option for long distance due to their extremely low losses of 0.1 dB/m [104]. In addition, with wavelength multiplexing, the capacity of a single silicon waveguide built using the SOI platform mentioned earlier, was demonstrated to exceed 1 Tbps using only a width of 520 nm [105].

#### 2.2.1. Towards Sub-100 nm Optical Interconnects

The recent trends indicate that the next move for optical interconnects integration is on-chip integration after board-level integration has reached a certain level of maturity [100,106,107]. As mentioned above, silicon waveguides with a width as low as 400 nm have been demonstrated, yet 400 nm is still much greater than today’s electronics interconnects dimensions, which are less than 100 nm when using the latest node technology [99]. Guiding the light inside the channels and waveguides is usually carried out by means of the total internal reflection (TIR) of light in a material with a high refractive index embedded inside another material with a lower refractive index [2]. The diffraction limit of TIR-based optical waveguides provides a lower limit to the acceptable dimension of a waveguide to successfully guide the light with a certain wavelength. To overcome the dimensions mismatch problem [100,101,102], both plasmonic waveguides, as well as photonic slot waveguides, offer possible solutions especially for the short range communication found in on-chip interconnects.

##### Plasmonic Waveguides

Plasmonic waveguides are based on the surface plasmon polariton (SPP) phenomenon, which occurs at the interface between a metal (or a heavily doped semiconductor) and a dielectric material. When coupling occurs between a photon incident on the interface and a free electron on the metal side, an oscillation occurs. Such oscillation is called SPP and can be confined in dimensions much smaller than the diffraction limit. However, the propagation losses of plasmonic waveguides are known to be very high due to the dissipation of SPP into the metal side. The propagation length of highly confined SPPs is so short that they cannot typically propagate more than ~1 cm. Although it was found that the energy per bit grew exponentially with the plasmonic interconnect length, it was also shown that plasmonic waveguides provided less crosstalk compared to the conventional interconnects [108]. Lengths beyond 100 µm for the plasmonic waveguide were found to be too lossy to be acceptable [109].

##### Slot Waveguide

A recently proposed waveguide that can be exploited to greatly minimize the silicon waveguide’s dimensions is the slot waveguide. Almeida et al. [110] proposed in 2004 the slot waveguide, which depends on another mechanism for guiding the light based on a discontinuity in the electric field at the interface between a region with a high index of refraction and another one with a low refractive index. Due to this discontinuity, the electric field would have a much higher intensity in the lower index region. Such a higher intensity would not decay if the low index region has a width that is approximately equal to or less than the decay length of the electric field. Slot waveguides can guide light in lower index regions as small as 50 nm; hence, it has the potential to be used in integrating optical interconnects into VLSI circuits [110]. The slot waveguide was proposed by Zhu et al. as a possible solution for the dimensions mismatch problem mentioned earlier [101].

The slot waveguide, proposed in 2004 [110], demonstrated that light can be confined in a 50 nm wide low index region while having a 20 times higher intensity compared to the conventional rectangular SiO_2_ waveguide. Using a slot waveguide to implement a ring resonator and a directional coupler was also demonstrated [111], which indicates that the slot waveguide can be successfully implemented in highly integrated photonics circuits.

Many attempts to extend the transmission range of the slot waveguide were carried out. For example, Xiang et al. [112] tried to combine the effect of electric field discontinuity at the low index region with the effect of a long-range surface plasmon polariton (SPP) wave [113] at the metal–low index interface. The result was a guided light mode that possessed a very long propagation range reaching 14.55 mm. The confinement of the light wave inside the low index region was also subwavelength confinement. The existence of silver strips, however, rendered the device incompatible with the CMOS fabrication process.

A slot waveguide with 1.5 dB/cm propagation loss at a 1.55 µm communication wavelength and 650 nm confinement of the electric field was proposed by Li et al. [114]. The material used was Indium gallium arsenide phosphide (InGaAsP), and it was Indium phosphide (InP) that showed that the slot waveguide fabricated from III–V semiconductor materials typically used in photonic integrated circuits (PICs) can have a low propagation loss.

**Figure 6:**
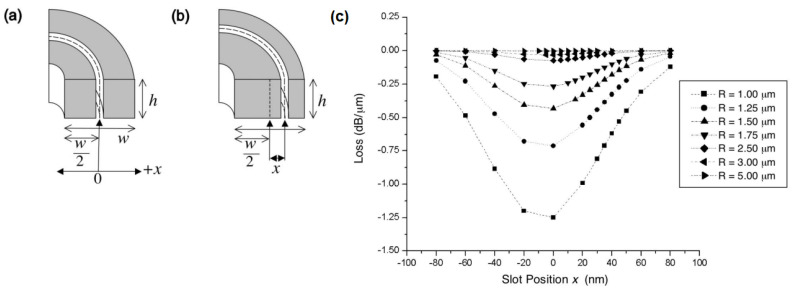
The waveguide structure with dimensions *h* = 250 nm and *w* = 450 nm: (**a**) The slot is centered in the waveguide and (**b**) the waveguide slot is offset by *x*. The positive *x* direction is towards the outside of the bend. (**c**) Slot wave-guide losses due to bending for different slot positions and bending radii. Reprinted by permission from the Optica Publishing Group [63].

The effective transmission over sharp bends in typical TIR-based optical interconnects, in general, poses a challenge since the light radiates outside the high index region at the sharp bend. Anderson et al. [63] investigated the preservation of the confinement within a low index region in a slot waveguide with sharp bends, as shown in Figure 6. The transmission efficiency from the bent sections in a slot interconnect network to straight sections can be affected and need to be studied. Ishizaka et al. investigated and proposed a solution to enhance the transmission efficiency from 33.8% to 84.2%, from bent sections to a straight section in a slot-based optical interconnect [115].

The investigation of the transmission effectiveness in a silicon slot waveguide with a 90-degree bend was conducted, and the recovery of such effectiveness from 55% to 95% was realized using simulation by increasing the excess free carriers in the silicon. The higher excess carriers dynamically convert the slot mode to the plasmonic mode [116].

The actual fabrication of the slot waveguide with 90-degree bends was investigated by Zhu et al. [101], showing measured bending losses of ~0.6–1.0 dB.

### 2.3. Modulators

As introduced earlier, there is a critical need for a higher bandwidth, lower power consumption, and lower thermal losses in the electronic circuit interconnects. While integrating the optical interconnects into electronic circuits might pose as the most likely direction to address such needs, optical modulators would play the critical part of converting the signal from electrical to optical and vice versa. Modulation can be performed either in a direct manner, whereby light is emitted from the laser light source only on a need basis by a trigger mechanism, or through an external manner whereby the laser light source continuously emits light while the modulator modulates the light wave characteristics based on the data to be encoded into the signal [117]. The external light modulation technique is found to provide better reliability, a higher speed, lower cost, and power budget [118].

Optical modulators can be classified as electro-absorption modulators, electro-refractive modulators, or thermo-optic modulators. In electro-refractive modulators, an electric field is applied to change the refractive index. In the case of electro-absorption modulators, the absorption coefficient is the characteristic that is undergoing change in the material due to the applied electric field, hence altering the material response to the incident light wave.

Electro-refractive modulators depend on the Pockels and the Kerr effects in the material to change the refractive index. The Pockels effect causes a linear change in the refractive index that corresponds to the change in the electric field. On the other hand, the change caused by the Kerr effect is proportional to the square of the electric field change [118]. A refractive modulator generally modulates the phase of the light beam by changing the refractive index, hence changing the speed or the phase velocity of the light in the material. Changing the speed would result in a phase change in the outgoing light wave leaving the modulator. Such a phase modulation can be converted to amplitude modulation using an interferometer or a directional coupler.

On the other hand, electro-absorption modulation depends on the Franz–Keldysh effect and the Quantum–Confined Stark effect (QCSE), which causes the band gap of the semiconductor material to be reduced when an electric field or a voltage is applied [118]. In the absence of an applied electric field, photons with energy lower than the semiconductor bandgap usually pass through the material, i.e., the material is transparent to them. However, once an electric field is applied and the bandgap is reduced, such photons will be absorbed causing an increase in the absorption coefficient of the semiconductor, which in return affects its refractive index due to the Kramers–Kronig relation [119].

The Pockels effect or linear electro-optic effect arises only in crystalline solids that lack inversion symmetry. Since silicon is a centrosymmetric crystal, it does not possess such an effect unless the inversion symmetry is broken [120]. Additionally, the Kerr effect, as well as the Franz–Keldysh effect, were found to be minimal in silicon, which limits the potential of electro-refraction and electro-absorption modulation using silicon in general [118,121]. On the other hand, a modulation based on the thermo-optic effect as well as the plasma dispersion effect can be successfully implemented using silicon.

Silicon has a large thermo-optic coefficient of $\frac{\Delta n}{\Delta T}=1.86\times 10^{-4}\;1/K$ [122]. This coefficient quantifies the expected change in the silicon refractive index due to a change in the temperature. Assuming the temperature is changed in a controllable manner and locally contained in the silicon waveguide, the experimental results showed that a 7 °C temperature change in a 500 µm long silicon device would result in a 180° phase shift and a change in a refractive index of approximately 1.3 × 10^−3^ [122].

The plasma dispersion effect changes both the real and imaginary parts of the refractive index by changing the free carrier’s density in silicon, hence affecting both the phase velocity and absorption of the material. Soref et al. [121] identified the change in the refractive index, Δn, and absorption coefficient, Δα, based on the density of the free carriers as follows:

(4) $$\Delta \alpha =\frac{e^{3}\lambda_{0}{}^{2}}{4\pi^{2}c^{3}\varepsilon_{0}n}\left(\frac{\Delta N_{e}}{\mu_{e}{\left(m_{ce}{}^{*}\right)}^{2}}+\frac{\Delta N_{h}}{\mu_{h}{\left(m_{ch}{}^{*}\right)}^{2}}\right)$$

(5) $$\Delta n=\frac{-e^{2}\lambda_{0}{}^{2}}{8\pi^{2}c^{2}\varepsilon_{0}n}\left(\frac{\Delta N_{e}}{m_{ce}{}^{*}}+\frac{\Delta N_{h}}{m_{ch}{}^{*}}\right)$$

where e is the electron charge, $\lambda_{0}$ is the free space wavelength, c is the speed of light, $\varepsilon_{0}$ is the permittivity of free space, n is the index of the unperturbed material, $\Delta N_{e}$/$\Delta N_{h}$ is the change in electron/hole carrier density, $m_{ce}{}^{*}$/$m_{ch}{}^{*}$ is the conductivity effective mass of electrons/holes, and $\mu_{e}$/$\mu_{h}$ is the electron/hole mobility. Although the refractive index change of silicon due to temperature change is comparable in magnitude to the refractive index change due to the plasma dispersion effect in silicon, the thermo-optic effect is much slower. Moreover, thermo-optic modulation can suffer from a bandwidth shift if the modulator is placed near thermal emissive electronic components, such as the CPU for example [118]. Several techniques have been proposed to alleviate this problem, such as using temperature-controlled heaters to negate the thermal effects of nearby components [123], or by placing other materials adjacent to silicon with thermo-optic coefficient that negates that of silicon [124,125]. Nonetheless, the latter approach will break the CMOS-compatibility. Hence, modulation based on plasma dispersion is preferred when using silicon.

Modulation based on plasma dispersion is however causing a change in both the phase shift and power of the light wave due to the fact that it affects both the real and imaginary parts of the refractive index. Additionally, the effect of the excess charge on the material refractive index is wavelength-dependent; therefore, designing a plasma dispersion-based modulator to meet certain operating conditions is less straightforward compared to the other modulation approaches in general.

#### 2.3.1. Modulators Performance Metrics

**Figure 7:**
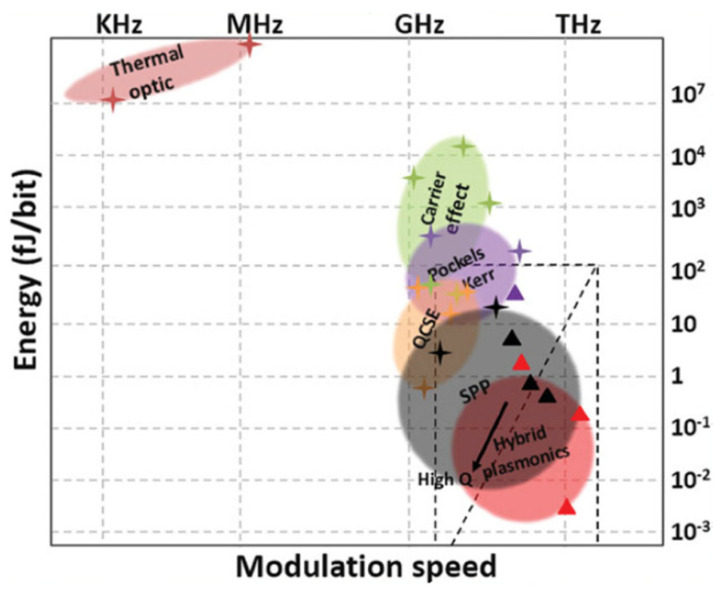
Speed versus energy consumption chart categorizes different types of electro-optical modulators [148], © 2022 by WILEY-VCH Verlag GmbH & Co. KGaA, Weinheim. Reprinted by permission from John Wiley and Sons: K. Liu, C. R. Ye, S. Khan, and V. J. Sorger, “Review and perspective on ultrafast wavelength-size electro-optic modulators,” Laser and Photonics Reviews, vol. 9, pp. 172–194, 2015.

Several performance metrics for modulators are jointly used to assess and compare their performance. The extinction ratio, energy per bit, footprint, modulation speed, optical bandwidth, insertion loss, and CMOS-compatibility are the main performance metrics used for electro-optic modulators in general. The extinction ratio represents the ratio between the on and off state of the modulator and is usually expressed in dB. It is considered as a helpful metric for judging the device’s immunity against noise. The optical or modulation bandwidth is the frequency range beyond which the optical power drops below 50%. The full width at half maximum (FWHM) is a common way to express such bandwidth. FWHM is the line width in nm or THz and represents the extent of the peak at 50% of its maximum power. The insertion loss represents the losses introduced into the overall system due to the insertion of the modulator in question into the system. The energy per bit is strongly dependent on the modulation speed and serves as an indication of the energy consumption of the modulator and its effect on the overall energy budget of the circuit into which the modulator is inserted. The modulation speed versus the energy per bit for various modulation approaches is shown below in Figure 7.

The thermo-optic modulation is the slowest and has the highest energy consumption, compared to other modulation techniques. Plasma dispersion modulation has a slightly higher energy budget and a comparable modulation speed compared to electro-refractive modulation. However, the energy consumption gap is higher when comparing the plasma dispersion modulation to the electro-absorption modulation and the plasmonic-based modulation techniques.

#### 2.3.2. Plasma Dispersion-Based Modulator Configurations

**Figure 8:**
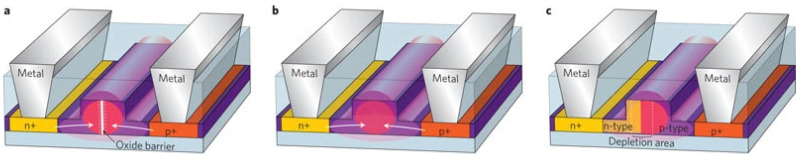
Cross sections of the typical plasma dispersion-based modulators configurations [118]. (**a**) Carrier accumulation in which a thin insulating SiO_2_ layer splits the waveguide and causes carriers to accumulate on both sides of the layer. (**b**) Carrier injection in which the waveguide is formed by an intrinsic layer separating a highly doped n and p regions. (**c**) Carrier depletion in which the lightly doped n and p regions are adjacent and form a PN junction in the waveguide itself. Reprinted by permission from Springer Nature: Nature Photonics “Silicon optical modulators,” G. T. Reed, G. Mashanovich, F. Y. Gardes, D. J. Thomson, © 2022.

**Figure 9:**
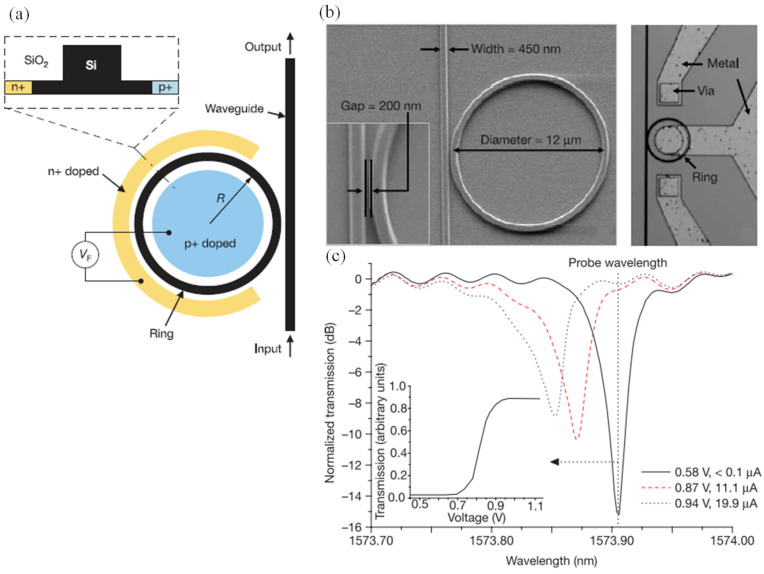
(**a**) Schematic layout of a carrier injection-based ring resonator modulator. (**b**) SEM and optical microscope top view images of the ring resonator. (**c**) The ring resonator’s transmission spectra for various bias voltage on the p-i-n structure. The inset in (**c**) shows the transfer function of the modulator [147]. Reprinted by permission from Springer Nature: Nature “Micrometre-scale silicon electro-optic modulator,” Q. Xu, B. Schmidt, S. Pradhan, M. Lipson, © 2022.

Different configurations for varying the carrier density in silicon to achieve high-speed modulation were extensively explored in the literature. Essentially, they were categorized as either carrier injection, carrier accumulation, or carrier depletion modulators (Figure 8). For the carrier injection modulation, much research was performed in such a category [121,126,127,128,129,130,131,132,133,134,135,136,137,138,139,140,141,142,143,144,145,146]. One of the early works in this area that serves as an adequate case study of carrier injection-based modulation was conducted by Xu et al. [147]. The device schematic is shown in Figure 9.

Many novel modulators that are based on carrier injection mechanism have been proposed [121,126,127,128,129,130,131,132,133,134,135,136,137,138,139,140,141,142,143,144,145,146,149]. However, a drawback in carrier injection-based modulators, in general, is the dependence of the modulation speed on the free carrier’s life time, which imposes a limit on the modulation speed and motivates the researchers to investigate other configurations of plasma dispersion modulation, such as carrier accumulation and carrier depletion.

**Figure 10:**
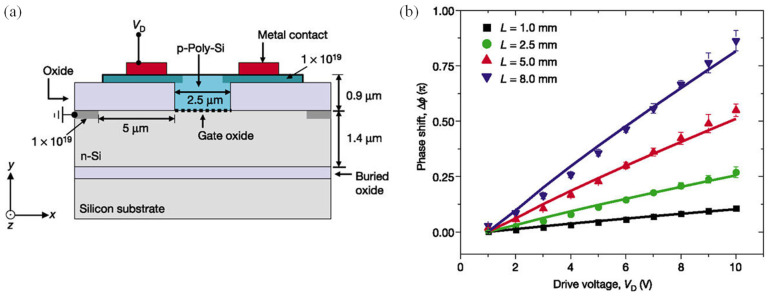
(**a**) Schematic layout of a carrier accumulation-based modulator. (**b**) Realized phase shift versus the drive voltage for different phase shifter length values [150]. Reprinted with permission from Springer Nature: Nature “A high-speed silicon optical modulator based on a metal–oxide–semiconductor capacitor,” A. Liu, R. Jones, L. Liao, D. Samara-Rubio, D. Rubin, O. Cohen, R. Nicolaescu, M. Paniccia, © 2022.

Research by Intel in 2004 sparked the use of carrier accumulation mechanisms in building plasma dispersion-based modulators [150]. In such research, a metal-oxide (MOS) capacitor was structured by placing a metal oxide sandwiched between p-poly-Si and an n-Si layers. The optical signal was confined in a rib structure formed by a variation in the oxide layer thickness, as shown in Figure 10. When applying a positive drive voltage, V_D_, to the p-type silicon layer and grounding the n-type layer, the carriers were accumulated on both sides of the thin gate oxide. Such an accumulation of charges caused a change in the charge density, which was related to the drive voltage by (6):

(6) $$\Delta N_{e}=\Delta N_{h}=\frac{\varepsilon_{0}\varepsilon_{r}}{et_{ox}t}\left(V_{D}-V_{FB}\right)$$

where $V_{FB}$ is the flat band voltage of the MOS capacitor. $\varepsilon_{0}$ and $\varepsilon_{r}$ are the vacuum permittivity and the low-frequency relative permittivity of the oxide, respectively. $t_{ox}$ and t are the gate oxide thickness and the effective charge layer thickness, respectively.

This device was able to reach a modulation frequency of more than 1 GHz in 2004 when all other carrier injection-based modulators, at that time, had a frequency in the range of tens of MHz. Generally, the modulation speed is higher in carrier accumulation-based modulators in comparison to other plasma dispersion modulation techniques, because the slow carrier generation and recombination process is not involved in carrier accumulation.

A higher modulation speed was later realized by subsequent research through further optimization [151,152,153,154]. A 3 dB bandwidth carrier accumulation-based modulator with a modulation frequency larger than 100 GHz was demonstrated [153].

The carrier depletion-based modulators not only offer the same speed advantage provided by the carrier accumulation modulators, but they also allow for easier fabrication due to the need for less complicated designs to achieve carrier depletion [155]. Many designs were proposed for the carrier depletion modulators. All of them can be categorized as being based on either the vertical PN junction, horizontal PN junction, or interleaved PN junction [155]. Carrier depletion modulators with 30, 40, and 50 Gbps were realized, yet some of the designs were lacking an adequate extinction ratio to be immune to possible signal noise [156,157,158,159,160,161,162,163,164]. A higher extinction ratio of 7.5 dB with a modulation speed of 50 Gbps was realized in 2014.

From the photonic device perspective, the electro-optic modulator requires either a resonator or a Mach–Zehnder interferometer (MZI) to convert refractive index perturbation into amplitude modulation. Due to the large device footprint of MZI devices, they are not suitable for on-chip communication applications. Additionally, resonator-based modulators showed a more efficient energy consumption per bit [118]. Several approaches were utilized for enhancing the light–matter interaction in micro-resonators and reaching an ultra-compact device footprint, while maintaining high speed and low energy consumption. Namely, micro-disks [154,165,166,167] , photonic crystal (PC) nanocavities [168,169,170,171,172], and micro rings [147,173,174,175,176,177].

Table 3 shows the Q-factor and energy per bit for resonator-based modulators reported in the literature. Only those built on the silicon photonic platform and derived by free carriers are shown. A general overview of the other types of modulators known to be incompatible with the CMOS fabrication process can be found in [178].

### 2.4. Photodetectors

Converting data back to the electrical domain was performed through photodetectors. Due to the silicon’s bandgap, the light at common telecom wavelengths, described roughly by the extended short wavelength infrared (e-SWIR) from 1 µm to 2.5 µm, did not have enough energy to generate an electrical current in the silicon. Again, we can observe a justification for integrating new materials, commonly III–V or germanium [183,184,185,186,187,188,189,190,191,192]. This section is organized as follows: first, we review the important metrics of a photodetector; then, we briefly explain the mechanism behind the primitive types of photodetectors, namely, p-i-n and avalanche, giving examples from literature for each type; and finally, we briefly discuss the recent advances and possible promising mechanisms.

#### 2.4.1. Performance Metrics

##### Responsivity

This parameter is very critical for the power consumption of the whole interconnect system. Quantum efficiency, η, is a measure of how much current is produced for each photon. A quantum efficiency of 100% means that each photon resulted in an electron that contributes to the generated current. Obviously, many factors can cause a low quantum efficiency, such as the recombination of the generated electron–hole pairs. On the other hand, as we shall soon see, there are ways to obtain a quantum efficiency exceeding 100%.

Responsivity, *R*, is a more common measure of the conversion efficiency from the optical domain to the electrical domain as given in (7)

(7) $$R=\frac{I}{P}$$

where *I* is the electrical current and *P* is the optical power. It is related to quantum efficiency by (8)

(8) $$R=\eta \frac{q\lambda }{hc}$$

where *q* is the charge of an electron, λ is the wavelength, h is Plank’s constant, and c is the speed of light.

Thus, we can also think of responsivity as the sensitivity. The higher the quantum efficiency, the more optical losses throughout the system can be acceptable without having to increase the laser power. A photodetector that is not sensitive will require a high-power laser and impose strict insertion loss requirements for the remaining components.

##### Speed

**Figure 11:**
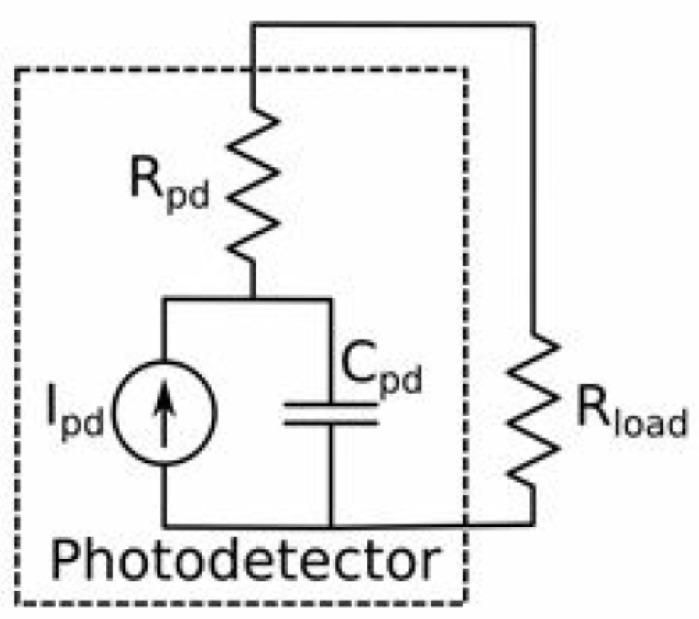
Photodetector circuit model [54], © Cambridge University Press 2015. Reprinted by permission from Cambridge University Press: L. Chrostowski and M. Hochberg, Silicon photonics design: from devices to systems.

Two factors are at play when considering the speed of a photodetector. First is the transit time: the time it takes for generated charge carriers to reach the contact and contribute to current. Therefore, it is the distance covered (geometry of the device) and the speed of charge carriers that determine the transit time. Mobility, and thus material choice plays an important role. The mobility of electrons in Germanium, for example, is more than double that in the silicon. The second is the RC response. The photodetector can be modeled as a current source ($I_{pd}$) with corresponding resistance $R_{pd}$ and capacitance $C_{pd}$, along with load impedance $R_{load}$, as shown in Figure 11. Several design factors affect the equivalent circuit parameters. For example, the capacitance $C_{pd}$ is affected by the depletion region width and the reverse bias voltage of photodiodes. The capacitance is inversely proportional to the depletion region width. One of the common approaches to reduce $C_{pd}$ is to introduce an intrinsic layer to increase the depletion region width, which would cause a reduction in the $C_{pd}$ value, resulting in increased speed of the photodiode. Additionally, the larger volume available for a photon to electron-hole conversion, in this case, would allow for higher quantum efficiency realization. The series resistance $R_{pd}$ is affected by the photodiode contacts and the type of the semiconductor material itself. A detailed account of the photodetectors’ equivalent circuits can be found in [193].

##### Dark Current

The dark current refers to a current that is generated without any light. Traditionally, this has been somewhat ignored as the electrical amplifier’s noise has dominated. However, there are now low-power electronics that are able to work with the low current generated by photodetectors and amplifiers are no longer necessary. It is thus becoming more important to discuss dark current as it contributes to noise.

In a photodetector with area, A, dark current, $I_{dark}$, is composed of bulk generated current, $J_{bulk}$, and surface generated current, $J_{surf}$, as given by (9):

(9) $$I_{dark}=J_{bulk}\cdot A+J_{surf}\sqrt{4\pi }\sqrt{A}$$

A main contributor to the bulk generated current is lattice defects, and it increases exponentially with increased bias voltage. The surface generated current is primarily due to dangling bonds, which are more difficult to take care of in Germanium than in silicon. Thus, the dark current increases with an increased bias voltage and with an increased photodetector area. In addition to the dark current, the shot noise, $I_{n}$, is also related to the bandwidth, BW, of the detector through (10), so that to increase the speed of a detector without degrading the signal-to-noise ratio, the dark current must be reduced.

(10) $$I_{n}=\sqrt{2qI_{dark}BW}$$

A common figure of merit used to evaluate photodetectors is the quantum efficiency-bandwidth product (η-BW). Table 4 compares the performance metrics of recent photodetectors that can be used in optical interconnect systems.

#### 2.4.2. Types of Photodetectors

A broad classification of infrared photodetectors divides them into thermal detectors and photon detectors. Thermal detectors depend on their operation of measuring changes in the temperature as an indication of the incident electromagnetic power. Although they can operate at room temperature, they are generally not suitable for high-speed applications. Photon detectors, on the other hand, depend on a change in the electrical properties caused by the absorption of photons. There are two types of photon detectors: photovoltaic detectors (PVs) and photoconductive (PC) detectors. While photoconductive detectors are based on the change in material conductivity due to the absorbed photons, PV detectors are based on the production of voltage across terminals due to absorbed photons and usually have a higher signal-to-noise ratio. As the detection of photons in PV detectors is directly dependent on the energy bandgap in comparison to the incident photon energy, different mechanisms were investigated in the literature to engineer a bandgap suitable for absorbing photons at the wavelength of interest. To illustrate the concept, a review of two primitive types of photodetectors is explained below (namely, the PIN photodetector and the avalanche photodetector).

##### PIN

The operational principle behind a p-type/intrinsic/n-type photodetector starts with light being absorbed at the intrinsic region, generating electron–hole pairs. It is thus important that the intrinsic region is made of a material with a bandgap that enables this process at the wavelength of operation. Crystalline silicon has a bandgap far larger than is required for absorption at telecom wavelengths (1.28 µm to 1.55 µm). As we will soon see, a common material of choice is Germanium. The p-type and n-type regions are required for the charge collection. It is imperative that the active region (in which photons are absorbed) is intrinsic as the dopants would cause the generated carriers to recombine.

The fabrication process described in [186] is typical for PIN germanium photodetectors. Using a 400 nm device layer on an SOI wafer, the n-type Si device layer is thinned down to 55 nm, on top of which a 340 nm thick layer of intrinsic Ge is selectively grown by CVD, followed by 90 nm p-type Ge and annealing. One contact is on the bottom n-type Si and the other is on the top p-type Ge. The electric field is applied vertically, and this type of photodetector is also known as the vertical PIN.

**Figure 12:**
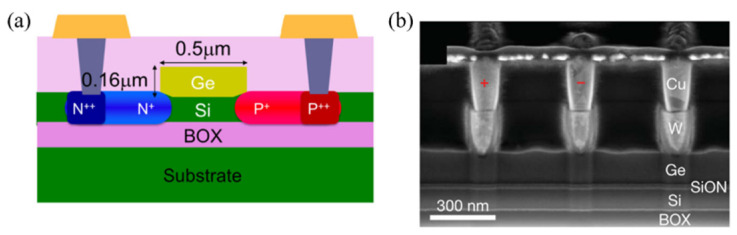
Different types of photodetectors. (**a**) Lateral PIN photodetector [194]. Reproduced with permission from Optica Publishing Group. (**b**) Lateral metal/semiconductor/metal avalanche photodetector [197]. Reprinted by permission from Springer Nature: Nature “Reinventing germanium avalanche photodetector for nanophotonic on-chip optical interconnects,” S. Assefa, F. Xia, Y. A. Vlasov, © 2022.

Figure 12 shows a lateral PIN, where the electric field is applied horizontally. It is a silicon-contacted Ge PIN photodetector in which a single intrinsic germanium layer not requiring a doped Ge layer is used, making the device more fabrication-friendly and streamlined with CMOS processing [194]. The metal contacts are on n-Si and p-Si silicon. By using a 160 nm thin Ge layer, the device had a low capacitance and demonstrated a high bandwidth of 67 GHz operating at −1 V. By moving the contact to the silicon, all the Ge surface was available to detect light, enabling a high responsivity of 0.93 A/W.

Mehta et al. proposed a polysilicon-based photodetector without any other material, such as Germanium [195]. The photodetector was based on creating defect states that enabled absorption even in the near-infrared range (i.e., especially at 1550 nm). The photodetector had a ring structure to enable efficient detection; otherwise, a very long structure would have to be used. The quantum efficiency was around 20% with a bandwidth of a few Gigahertz. Such a design enabled a completely CMOS-compatible photonic integrated circuit.

##### Avalanche

In an avalanche photodetector (APD), the electron-hole pairs generated cause additional generation of the electron-hole pairs through what is known as an avalanche process. Thus, the quantum efficiency can exceed 100%. The gain of an APD is thus quite important. APDs can become quite noisy because of the gain process and there are interesting ways to overcome the dark current.

One approach is to build upon a PIN photodetector, such as a vertical PIN photodetector with an avalanche amplification [198]. Here, a low-voltage germanium waveguide avalanche photodetector (APD) with a gain × bandwidth product above 100 GHz was demonstrated. The device was fabricated on IMEC fully integrated silicon photonics platform.

The approach taken by Assefa et al. was based on metal/semiconductor/metal, where the electric field was applied laterally [197]. A germanium layer was used for the detection while a separate silicon layer was used for the amplification. Instead of the epitaxial growth of the Ge layer, which is normally polycrystalline, Assefa et al. opted for rapid melting growth, allowing a defect-free single-crystal Ge layer that was, only 140 nm as shown in Figure 12b. 70% noise reduction was fulfilled by applying a strong non-uniform electric field.

#### 2.4.3. Recent Advances

Generally, compounds, such as InGaAs and GaInAsSb, produce a suitable bandgap for photo detection; however, lattice mismatch causes a significant increase in the dark current. A Hg_1−x_Cd_x_Te alloy can mitigate this problem to a certain degree, since HgTe and CdTe have a very close lattice constant. However, detectors made from HgCdTe suffer from poor material uniformity, low yield, and surface instability [202,203]. Quantum structures, such as the type II super lattice (T2SL) and type II multiple quantum wells (MQWs) with band alignment, possess a narrower effective bandgap than their individual constituent materials, offering an opportunity for infrared photo detection with a lower dark current compared to HgCdTe-based detectors [204]. Some PC detectors, such as quantum well infrared photodetectors (QWIPs), in which the device resistance changes under illumination, are considered as better alternatives to HgCdTe since they have better material uniformity [205]. A detailed review of the recent advances in photodetector research for the infrared spectrum was given by Chen et al. [206]. Recent interesting research stands out for being able to achieve a ultra-high bandwidth of 265 GHz with a low dark current of 100 nA and a high responsivity of 0.3 A/W, hence outperforming most sophisticated InP photodetectors in terms of high-speed performance, while being made from Ge and silicon, making it more suitable for integration with electronics circuits [196].

## 3. Complete Interconnect Systems

**Figure 13:**
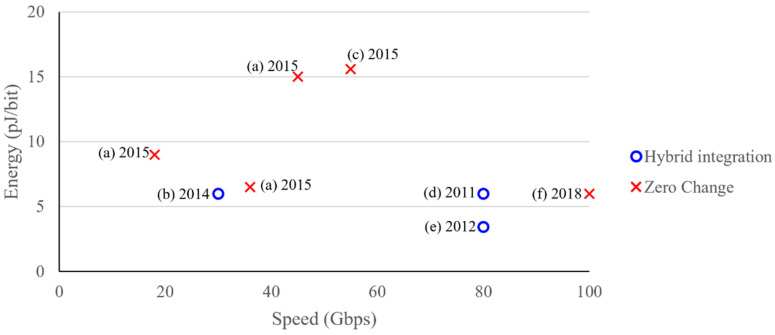
Summary of the various recent complete interconnect systems. (**a**) Three cases based on 180 nm CMOS technology using a bulk silicon wafer. All-optical devices based on polysilicon. Each channel has a maximum of 5 Gbps; the reported speed is with WDM [49]. (**b**) Two-chip approach: optical chip built using III–V materials, integrated with an electronic chip built using 32 nm SOI CMOS technology. A reported speed of 30 Gbps refers to a single-channel [219]. (**c**) A zero-change approach based on a 45 nm SOI CMOS process that includes SiGe, demonstrated only one wavelength operation at 5 Gbps; the reported speed of 55 Gbps is the potential bandwidth if WDM is implemented [70]. (**d**) Eight 10 Gbps WDM channels, for a total speed of 80 Gbps. A 130 nm SOI was used for the optical devices, and a 40 nm CMOS was used for the electronic devices. The reported power consumption excludes ring tuning and laser power [217]. (**e**) A similar approach to (**d**), with a more efficient thermal tuning of modulators [218]. (**f**) A zero-change approach based on a 65 nm CMOS technology using a bulk silicon wafer, demonstrated only one wavelength operation at 10 Gbps and 600 fJ/bit power consumption. Reported speed and power consumption are achievable if WDM is implemented [220].

While a plethora of components has been successfully demonstrated, the real challenge is integrating the different components together to arrive at a complete interconnect system. The first serious suggestions of an optical interconnect to electronic chips discussed interfacing to and from CMOS chips, but shied away from the actual integration [207]. CMOS-compatible optical interconnects were suggested a decade ago [99,208,209], and, since then, several roadmaps have been steering the research directions to make sure that future optical interconnects can be integrated with electronics chips [33,99,208]. The most recent of these roadmaps deserve to be highlighted due to their comprehensive approach to and involvement of many key players in academia and industry [55,210]. One of the key requirements was low power consumption, and Miller recently argued that we should target 10 fJ/bit [211]. The objective of this section is to review the progress and compare it with these roadmaps, summarized in Figure 13. In addition, we see how rearranging interconnects from a systems-level point of view using the current devices can be very beneficial.

As we demonstrated at length, there is a plethora of device designs for the different components of the interconnect system. However, few solutions exist that actually bring all these components together to demonstrate a working fully integrated system. The industry has a strong role here, with some companies focusing on silicon photonics, such as Luxtera and Aurrion, and other large players being involved, such as Intel, Sun Microsystems, and later Oracle, IBM, and HP. We see that in most cases, some forms of partnership is often important, whether companies cooperate with each other or collaborate with academia.

Any review of CMOS-compatible optical interconnects must give credit to Luxtera’s efforts. While they demonstrated a system capable of 10 Gbps, they laid out their views on reaching 100 Gbps and 1 Tbps, and they even discussed the potential of reaching speeds of 10 Tbps [71].

The group at the University of California Santa Barbara spearheaded the hybrid integration research efforts, especially with regard to integrating III–V lasers on silicon; in fact, some of the work on optical interconnects by Intel was conducted in collaboration with UCSB [70,86,93]. The research finally culminated in the development of a whopping 2.56 Tbps optical interconnect, although the experimental demonstration used only one channel (40 Gbps) [212]. Intel started working on silicon photonics components over a decade ago [76,77,86,150,157,158,213], and when they announced their 50 Gbps system in 2010 [214,215], it was a real attention grabber.

Sun Microsystems/Oracle also published their optical interconnect components [69], followed by complete systems reaching speeds of 80 Gbps [216,217,218]. The approach made use of a 40 nm CMOS for the electrical components and 130 nm SOI for the optical devices, in addition to the Ge photodiode, giving detailed accounts of feasible integration of the dies. This approach was extremely efficient, being the first interconnect system to consume energy less than 1 pJ/bit (ring tuning and laser power excluded).

IBM and Aurrion joined forces to demonstrate a 30 Gbps optical interconnect system with a power efficiency of 3 pJ/bit (excluding laser power) [219]. The optical components were fabricated using III–V material, integrated with a low power 32 nm CMOS electronics driving circuitry.

**Figure 14:**
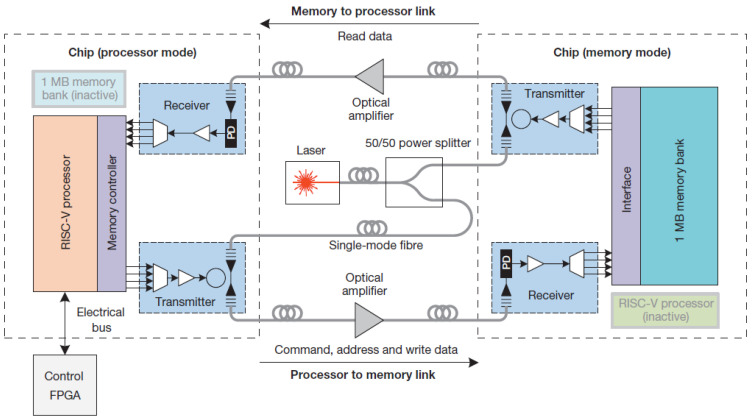
Silicon photonics optical interconnect co-integrated with electronics to provide communication between the processor and memory [70]. Reprinted by permission from Springer Nature: Nature “ Single-chip microprocessor that communicates directly using light,” C. Sun, M. T. Wade, Y. Lee, J. S. Orcutt, L. Alloatti, M. S. Georgas, A. S. Waterman, J. M. Shainline, R. R. Avizienis, S. Lin, B. R. Moss, R. Kumar, F. Pavanello, A. H. Atabaki, H. M. Cook, A. J. Ou, J. C. Leu, Y.-H. Chen, K. Asanović, R. J. Ram, M. A. Popović, and V. M. Stojanović, © 2022.

A strong collaboration between the research groups in MIT, the University of Colorado, and the University of California at Berkeley is driving the efforts for zero-change integration, and we already discussed their photodetectors [195] and modulators earlier on in the paper [123]. Sun et al. led a fantastic demonstration of a microprocessor communicating with memory optically [70], as shown in Figure 14. They selected to work with a 1180 nm wavelength instead of the standard 1300 nm and 1550 nm wavelengths, enabling working with a SiGe photodetector. SiGe was used in advanced the CMOS processes.

While all the previously mentioned interconnect systems used Ge for photodetectors, which are still considered as a custom platform, this group used their Ge-free photodetector that was explained earlier using a p-i-n resonator structure that relied on the defect states [195]. This lead to several zero-change electronics–photonics devices over the past few years [48]. This approach resulted in an optical link of 5 Gbps speed with a 15 pJ/bit efficiency [49]. While this was somewhat a slower and less efficient optical interconnect system than the previously discussed interconnect, the zero-change integration made adoption side-by-side electronics much more realistic. Most importantly, the process in [49] uses bulk silicon wafers (not SOI), making it much more economically viable than the interconnects that rely on SOI wafers, such as the zero-change interconnect in [70,221], as well as all the hybrid integration interconnects.

More recently, Atabaki et al. integrated photonics with silicon nanoelectronics using 65 nm CMOS technology (not SOI), and demonstrated optical transceivers capable of 10 Gbps per channel [220,222].

## 4. Conclusions

In conclusion, the metal interconnects in today’s integrated circuit technology are facing challenges in meeting future target performance with further downscaling in dimensions, and the adaptation of the many-core approach to maintain Moore’s law. The future trend of many-cores in particular has most of its power budget in the design consumed in metal interconnects to facilitate the communication among different cores, despite using state-of-the-art interconnect optimization and power management techniques. The bandwidth requirement for many-core designs is expected to reach 100 Gbps, causing metal interconnects to be a serious bottleneck in the future of VLSI chips [1,2,3,5,6]. Optical interconnects seem to be a viable alternative that can come to the rescue and substitute metal interconnects, achieving future target bandwidth and power consumption limits. On-chip light sources in a typical optical interconnect system are preferred over external sources, due to the higher coupling losses introduced by external sources and the post-fabrication processing needed by the butt coupler used to couple light from external sources to a wideband optical interconnect systems. Silicon-based on-chip laser sources are not typically available because silicon is an indirect band gap semiconductor. The heterogeneous integration with III–V on-chip sources is promising, yet, to date, it is not possible without applying some changes to the currently used fabrication flow to build integrated silicon photonics circuits. Using the quantum confinement effect, silicon can emit light. Many research articles investigated building silicon lasers from 0D and 1D structures to emit light at cryogenic temperature in order to mitigate the heat produced by the phonons generated in the same process. A nanocrystal-based silicon laser was realized at room temperature, based on the amplification of spontaneous emission using an external light source. Raman scattering was also utilized to produce a lasing of 50 mW power and efficiency of around 28%. Laser sources based on III–V semiconductors have superior performance compared to silicon due to its direct bandgap. A structure composed of InAs quantum dots grown on silicon was able to generate a laser in a temperature of up to 110 °C, thus they are compatible with the harsh environment often found in electronic ICs. The realized efficiency was 37% and output power was above 176 mW, which greatly outperformed the Si-based lasers. The heterogeneous integration of III–V with silicon was found to produce superior results compared to using III–V material alone, since the free carrier generated in III–V due to spontaneous emission was swept away to the Si layer without attenuating the gain. The line width of the laser source was of particular importance for wavelength division multiplexing. Recent research shows that a 1 Tbps hybrid silicon photonic transmitter can be realized using 25 laser sources. In regard to the optical waveguide, while it offers a better propagation delay than its electrical counterpart, the required dimensions for a typical optical waveguide, which guide the light based on the total internal reflection inside the high index core, is considerably larger than its electrical counterpart due to the diffraction limit and light wavelength being larger than 1 micron. Additionally, due to the large size of the guided light mode footprint, a relatively large separation was required among adjacent waveguides. A single-mode silicon strip waveguide, 220 nm in height and widths of 400 nm to 500 nm with losses of around 0.1 to 0.3 dB/mm, was realized. Lower losses can be attained as well using silicon nitride. Special attention is given to the slot waveguide that can confine and guide visible and near-IR light in a sub100 nm low index region surrounded by higher index regions. To increase the efficiency of transmission over 90-degree bends in silicon slot waveguides, from 55% to 95%, a conversion of the dielectric slot mode into a plasmonic slot mode is realized through free carrier generation in the silicon region of the waveguide. For modulators, external modulators, which depend on a constant power laser source continuously generating light that is being modulated using a modulator component, have a higher speed, lower cost, and lower power consumption compared to the direct modulators. Since, at the standard communication wavelengths, silicon lacks the Pockels and Kerr effects required for electro-refraction modulation, the electro-refraction modulation technique cannot be exploited in silicon. Although the thermo-optic effect was high in silicon and can be utilized for modulation, it provided a low modulation speed. Silicon also lacks electro-absorption effects, such as Franz–Keldysh, and quantum confined stark effects (QCSE). Many researchers tried to create a silicon modulator by inducing free carriers through doping, which would affect both the refractive index and the absorption of the material; however, the speed of carrier injection-based silicon modulators were generally limited compared to the other types of modulators. This was because of the dependence on the free-carrier lifetime. The carrier accumulation mechanism was utilized in silicon to overcome the speed limitation of carrier injection silicon modulators. However, the fabrication was much more difficult for the carrier accumulation-based silicon modulators compared to the carrier injection modulators. A promising approach is using carrier-depletion in silicon modulators, which is easy to fabricate and can offer high-speed modulation. A speed higher than 100 GHz was demonstrated by engineering the intrinsic resistance and capacitance of an optical modulator that could be built to be CMOS-compatible [152]. Resonator-based Si modulators were found in the literature to be more energy-efficient while maintaining a much smaller footprint than the MZI-based silicon modulators. Using silicon for photodetectors posed a problem due to its transmission capability in the near- and mid-IR spectrums, which made it more suitable for building waveguides for transmission rather than photodetectors. The III–V materials or Ge could be effectively used to build suitable photodetectors. Additionally, adding Ge or III–V defects to silicon altered the optical response in the desired photo-detection range and enabled building silicon photodetectors with an acceptable performance [188,189,190,191]. Responsivity, speed, and the dark current were critical performance metrics for the photodetectors that were discussed and used in this article to compare various on-chip photodetectors, as shown in Table 4. The p-i-n photodetectors, for which germanium was used as the intrinsic layer for its low bandgap energy, were discussed. The examples of vertical and lateral variations of germanium-based p-i-n photodetectors were shown. A breakthrough in silicon-based photodetectors occurred in 2014, [198] when a photo-detector made from poly-silicon only contained defect states that caused absorption even in the near-IR range. The quantum efficiency was around 20% and a bandwidth of few GHz was realized. With that realization, it was possible to build a completely CMOS-compatible optical interconnect system with zero-change in the current CMOS fabrication flow. The efforts to build p-i-p photodetectors that utilized the avalanche effect to achieve a quantum efficiency larger than 100% were also surveyed. Recent interesting research stands out for being able to achieve an ultra-high bandwidth of 265 GHz with a low dark current (100 nA) and a high responsivity of 0.3 A/W, hence outperforming the most sophisticated InP photodetectors in terms of its high-speed performance, while being made from Ge and silicon, making it more suitable for the integration with electronics circuits [196]. A summary of the complete interconnect systems built by academia and the industry was presented and showed speeds ranging from 20 Gbps to 80 Gbps and energy consumption from 50 pJ/bit down to 1 pJ/bit. Major road blockers were removed from the way towards the realization of a mass-produced optical interconnect system integrated into everyday electronics. By focusing on the design aspects of a complete optical interconnect, a focus that is similar to that being received by individual optical components in current research, such a realization would be reached sooner.

## Figures and Tables

**Table 1:** Insertion loss and bandwidth for the different types of fiber-to-waveguide couplers [58]. Adapted under CC BY.

Coupler Type	Insertion Loss	1 dB Bandwidth	1 dB Alignment Tolerance
1D grating coupler [59]	1.6 dB	40 nm	±2.5 µm
1D grating coupler [60]	1.6 dB	40 nm	±10 µm
2D Ggating coupler [61]	3.2 dB	35 nm	±2.5 µm
Edge coupler [57]	1.2 dB	200 nm TE 150 nm TM	±500 nm
Evanescent coupler [62]	1.0 dB	>>40 nm	>±2.5 µm

**Table 2:** Summary of various on-chip sources.

Work	External Laser Needed	Operation Temperature	Size	Efficiency	Maximum Output Power	Linewidth
Si cone-shaped pores [74]	Yes	10–80 K		0.001%		
Si CW Raman laser [77]	Yes	25 °C	4.8 cm long cavity	9.4%		
Si pulsed Raman laser [76]	Yes	25 °C	4.8 cm long cavity	28%		
InAs QDs on Si [82]	No	20–119 °C		37%		
InAs/GaAs QDs on Si [84]	No	20–120 °C			105 mW	
InAs QDs on GaAs on Si [83]	No	20–85 °C	6 × 1341 µm^2^	31%	185 mW	
AlGaInAs quantum wells on Si [86]	No	15–40 °C	860 µm long cavity	12.7%	1.8 mW	
Hybrid microring [87]			20 to 50 µm diameter ring	0.25–0.53%		
AlGaInAs quantum wells on Si with diffraction grating [88]	No	25 °C		5%	2.3 mW	
Ring resonator Si/InP [89]	No	20 °C	0.81 mm^2^		3 mW to 10 mW	220 Hz to 2 kHz
InP amplifier/Si_3_N_4_ waveguide circuit [90]	No	25 °C			23 mW	40 Hz
InAlGaAs multiple quantum well (MQW)/InP/Si/SiN extended-distributed Bragg reflector (E-DBR) [91]	No	20–75 °C	1.5 to 2.5 mm gain section		10 to 25 mW	1 kHz to 400 Hz 3 Hz (w/ultra-high-Q SiN photonics)
III–V distributed feedback (DFB) laser coupled to CMOS-ready ultra-high-Q Si_3_N_4_ microresonator [92]	No					1.2 Hz

**Table 3:** Parameters for the silicon resonator-based modulators [165,168,173,179,180,181,182].

Reference	Resonator/Capacitor Type	Q-Factor	E_bit_ (fJ/bit)
[168]	PC nanocavity/ITO MOS	3700	3.25
[165]	Micro-disk/vertical PN junction	6480	1
[180]	Micro-ring/interleaved PN junction	14,500	66
[181]	Micro-ring/Si/oxide/Si MOS	3500	180
[179]	Micro-disk/vertical PN junction	9700	61
[182]	Micro-ring/lateral PN junction	14,500	50

**Table 4:** Summary of various on-chip photodetectors.

Type	Technology	Operational Wavelength	−3 dB Bandwidth	Responsivity	Dark Current	*η*-BW (GHz)
PIN	Ge/SOI [186]	1.55 µm	42 GHz	1 A/W	18 nA	33.6
	Ge/130 nm SOI/90 nm CMOS [69]	1.55 µm	10 GHz	0.7 A/W	3 µA	5.6
	Si-LPIN GePD [194]	1.3 µm 1.55 µm	44 GHz 67 GHz	0.93 A/W 0.74 A/W	4 nA	39 39.7
	CMOS [195]	1.28 µm 1.55 µm	8 GHz 4 GHz	0.2 A/W 0.25 A/W	50 pA	1.6 0.8
	Ge-Fin [196]	1.55 µm	265 GHz	0.3 A/W	100 nA	86
Avalanche	Ge M-S-M [197]	1.3 µm 1.55 µm	40 GHz	0.4 A/W 0.14 A/W	50 µA	15.3 4.5
	VPIN Ge [198]	1.55 µm	10 GHz	0.6 A/W	17 nA	4.8
	Defect-mediated Si [199]	1.96 µm	12.5 GHz	0.3 A/W	1 µA	
MQW	GeSn MQWs on Si [200]	2 µm	1.2 GHz	0.023 A/W	0.031 A	
	GeSn MQWs on Si [201]	1.55 µm to 2 µm	10 GHz	0.2 at 1.55 µm	44 mA	

LPIN = lateral p-type/intrinsic/n-type; VPIN = vertical p-type/intrinsic/n-type; MSM = metal/semiconductor/metal; and MQW = multiple quantum well.
